# Effects of Stress and Allopregnanolone on the Expression of Neurotrophins and TrkB Receptor in the Sheep Hippocampus

**DOI:** 10.3390/ijms26136190

**Published:** 2025-06-27

**Authors:** Tomasz Misztal, Patrycja Młotkowska, Elżbieta Marciniak, Marcin Barszcz, Bartosz Osuch, Alina Gajewska, Anna Misztal

**Affiliations:** The Kielanowski Institute of Animal Physiology and Nutrition, Polish Academy of Sciences, Instytucka 3 Str., 05-110 Jabłonna, Poland; p.mlotkowska@ifzz.pl (P.M.); e.marciniak@ifzz.pl (E.M.); m.barszcz@ifzz.pl (M.B.); b.osuch@ifzz.pl (B.O.); a.gajewska@ifzz.pl (A.G.); a.misztal@ifzz.pl (A.M.)

**Keywords:** allopregnanolone, neurotrophins, BDNF, TrkB, hippocampus, sheep

## Abstract

Neurotrophins, such as brain-derived neurotrophic factor (BDNF) and neurosteroids, including allopregnanolone (ALLO), play critical roles in modulating neuronal activity in the brain. Levels of these compounds dynamically fluctuate in response to physiological and environmental conditions, particularly stress, suggesting complex regulatory interactions. This study aimed to explore the effects of acute stress and ALLO (individually and combined) on hippocampal expression of BDNF, its TrkB receptor, and other neurotrophins in sheep, a translational large animal model. Adult, luteal-phase sheep (n = 24), implanted with a guide cannula into the third brain ventricle, were divided into four experimental groups: (i) 3 days of Ringer–Locke solution (RL) infusion as the control; (ii) 3 days of RL infusion with 4 h acute stress on day three; (iii) 3 days of ALLO infusion (4 × 15 µg/60 µL/30 min) with 4 h acute stress on day three; and (iv) 3 days of ALLO infusion alone (n = 6 per group). Both acute stress and ALLO alone significantly reduced BDNF concentration and *BDNF* transcript abundance in the hippocampal CA1 and CA3 fields compared to the control group. The combined application of both stress and ALLO resulted in decreased levels of these parameters, except for BDNF concentration in the CA3 region. Additionally, TrkB mRNA expression in both hippocampal fields was significantly reduced in all treatment groups. Changes in mRNA levels for other neurotrophins, including nerve growth factor (NGF) and neurotrophin 3 (NT3) and 4 (NT4), varied under experimental conditions. While an inhibitory effect was predominant, NGF expression in the CA1 region remained unaffected by stress or ALLO. Interestingly, stress alone induced a significant increase in NT4 mRNA expression in the CA3 field compared to the control. In conclusion, the study demonstrated that a 4 h acute stress exposure inhibited the synthesis of BDNF, TrkB, and several other neurotrophins in the sheep hippocampus. Furthermore, ALLO, whose increased levels are highly correlated with the initial stress response, may serve as a mediator of this stress effect, temporarily preventing over-stimulation of hippocampal BDNF release and signaling.

## 1. Introduction

The mammalian brain is not merely a recipient of steroid hormone signals but also has the intrinsic capacity to produce and metabolize such compounds. Specific enzyme systems responsible for steroid synthesis have been identified in various brain structures, including neurons and glial cells [1,2]. These locally synthesized neurosteroids function as neuromodulators and paracrine signals, with their concentrations varying dynamically during neural development, stress responses, pregnancy, and aging processes [3]. Importantly, disruptions in neurosteroidogenesis have been associated with a range of behavioral and neurological disorders and, in certain cases, with the development of neurodegenerative diseases [4]. Among these neuroprotective compounds, allopregnanolone (ALLO), a progesterone derivative, represents a particularly significant molecule. Unlike its precursor, ALLO does not act through nuclear receptors; instead, it modulates the brain’s principal inhibitory neurotransmitter, gamma-aminobutyric acid (GABA) [5]. By activating GABA_A_ receptor subunit, ALLO exerts anxiolytic and anti-stress effects and modulates diverse neuronal functions [6,7,8]. The therapeutic effects of this compound have been further demonstrated in experimental models of Alzheimer’s disease [9] and Parkinson’s disease [10].

Neurotrophins represent a crucial family of peptides that play fundamental roles in nervous system development and various physiological and pathological processes in the mammalian brain. These proteins regulate synaptic plasticity, neurotransmission, and support the survival and health of specific neuronal populations [11,12]. The neurotrophin family includes brain-derived neurotrophic factor (BDNF), nerve growth factor (NGF), neurotrophin 3 (NT3), and neurotrophin 4 (NT4), which exert their effects through several types of tropomyosin receptor kinases (Trk), such as TrkA, TrkB, and TrkC, as well as the low affinity P75 neurotrophin receptor (p75NTR). These receptors can function independently or through complex interactions, enabling them to mediate a diverse array of biological functions [11,12]. BDNF is the most abundant and active neurotrophin in the brain, widely regarded as a critical survival and growth factor for CNS neurons. This peptide is particularly concentrated in hippocampal neurons, a key limbic structure responsible for long-term memory formation, spatial navigation, and emotional processing [13,14]. Similarly to other neurotrophins, BDNF is initially synthesized as a precursor molecule (pro-BDNF), with both pro-BDNF and mature BDNF frequently co-existing and being co-released in many brain regions. However, the quantitative contribution of these two forms under physiological and pathological conditions remains an active area of investigation [14]. Interestingly, pro-BDNF and mature BDNF affect neuronal activity by binding to distinct receptors, i.e., pro-BDNF to p75NTR and mature BDNF to TrkB, resulting in opposing effects on neuronal function [11,12,13,14].

The secretion of BDNF is closely linked to neuronal excitation, which is controlled by a variety of stimuli and proteins that elevate cytoplasmic Ca^2+^ levels [15]. In the mammalian brain, excitatory transmission is mainly mediated by the neurotransmitter glutamate (Glu) through its ionotropic and metabotropic receptors [16]. The interaction between Glu and BDNF influences neuronal proliferation and other neuronal commitments, acting as a positive regulator of neurogenesis in both the developing and adult brain [17]. Homeostatic neuronal excitation and the associated BDNF signaling can be affected by various environmental factors. While exercise, dietary energy restriction, and cognitive stimulation increase BDNF levels in the hippocampus, adverse conditions including chronic stress, depression, and diabetes suppress BDNF production [18]. From the perspective of our study, it is important to note that different types of stress and its duration can differentially affect BDNF levels in specific brain areas [19]. Considering that acute stress is closely correlated with increased ALLO levels in the CNS [20,21], and that ALLO primarily modulates neuronal activity through GABAergic inhibition [22], this neurosteroid likely participates in stress-induced regulation of BDNF signaling [22]. The relationship between ALLO and BDNF has been implicated in various behavioral and neurological conditions, though the nature of their interaction remains poorly understood [23]. To address this knowledge gap, the present study aimed to explore the effects of acute stressors and ALLO, both independently and in combination, on BDNF synthesis and the expression of its TrkB receptor, and other neurotrophins in the hippocampus of the sheep model.

## 2. Results

### 2.1. BDNF Expression in the CA1 and CA3 Regions

**Figure 1:**
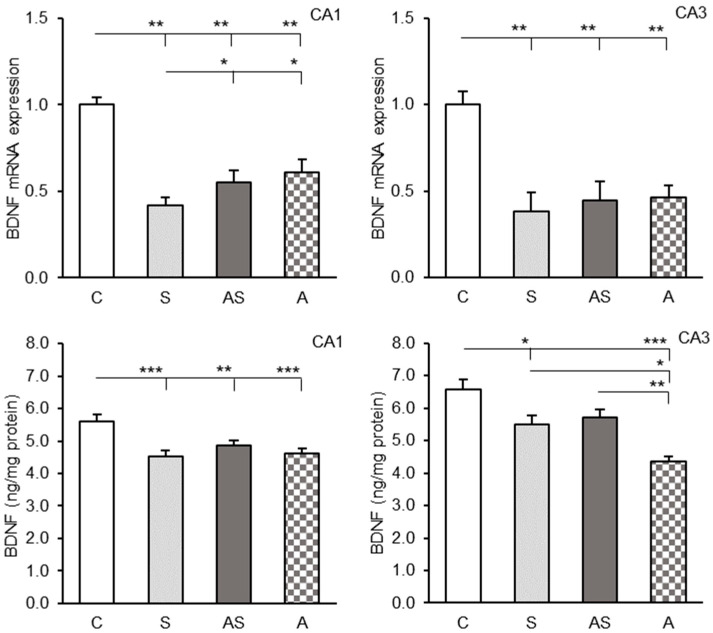
Relative brain derived neurotrophic factor (BDNF) mRNA expression (mean ± SEM, (**top**)) and BDNF protein levels (mean ± SEM, (**bottom**)) in hippocampal CA1 (**left panel**) and CA3 (**right panel**) fields of sheep treated with control solution (C), control solution and stressful stimuli (S), allopregnanolone (4 × 15 μg/60 μL/30 min/day) and stressful stimuli (AS) and allopregnanolone alone (A). Significance of differences: * *p* < 0.05, ** *p* < 0.01, *** *p* < 001.

Strong BDNF mRNA expression was observed in both the CA1 and CA3 hippocampal fields demonstrating a trend towards higher levels in the CA1 region. All experimental factors significantly reduced *BDNF* gene expression (*p* < 0.01) in both hippocampal fields compared to the control group (Figure 1, top). Moreover, *BDNF* transcript levels in the CA1 region were lower (*p* < 0.05) in the group exposed to stress alone (S) compared to sheep infused with ALLO alone (A) or those receiving ALLO combined with stress exposure (AS). No significant differences in *BDNF* transcript levels were observed between the experimental groups in the CA3 field.

Both stress-only (S) and ALLO-only (A) groups showed highly significant reductions in CA1 BDNF protein levels compared to controls (*p* < 0.001), (Figure 1, bottom, left panel). The stress + ALLO group (AS) also showed significantly lower BDNF concentrations (*p* < 0.01), though this decrease was less pronounced. No significant differences were observed between the experimental groups (S, A, AS).

A decrease in BDNF protein levels was also observed in the hippocampal CA3 region in the groups of sheep subjected to stress (S, *p* < 0.05) and those infused with ALLO alone (A, *p* < 0.001), (Figure 1, bottom, right panel) compared to the control. Interestingly, no significant differences were found between the controls and the sheep exposed to stress combined with ALLO infusion (AS). Additionally, BDNF levels in sheep infused with ALLO alone (A) were significantly lower than in the stressed sheep (S, *p* < 0.05) and the sheep exposed to stress in combination with ALLO infusion (AS, *p* < 0.01).

### 2.2. TrkB mRNA Levels in the CA1 and CA3 Fields

**Figure 2:**
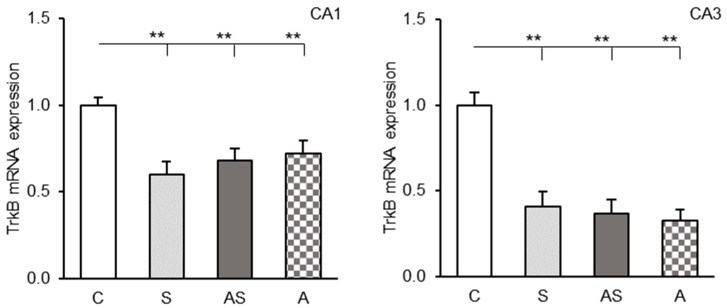
Relative tropomyosin receptor kinase B (TrkB) mRNA expression (mean ± SEM, (**top**)) in hippocampal CA1 (**left panel**) and CA3 (**right panel**) fields of sheep treated with control solution (C), control solution and stressful stimuli (S), allopregnanolone (4 × 15 μg/60 μL/ 30min/day) and stressful stimuli (AS), and allopregnanolone alone (A). Significance of differences: ** *p* < 0.01.

Control animals showed strong TrkB receptor mRNA expression in both the CA1 and CA3 regions of the hippocampus. All experimental groups (stress-only, ALLO-only, and stress + ALLO) exhibited significantly lower TrkB mRNA levels compared to controls (*p* < 0.01), (Figure 2). No significant differences in *TrkB* gene expression were found between experimental groups in either hippocampal field.

### 2.3. Expression Profiles of NGF, NT3 and NT4 Transcripts in the CA1 and CA3 Fields

The expression of three neurotrophin mRNAs (NGF, NT3, NT4) was detected in both CA1 and CA3 hippocampal regions, showing varying intensity. Experimental treatments induced differential changes in transcript abundance for these neurotrophins in the hippocampal fields.

**Figure 3:**
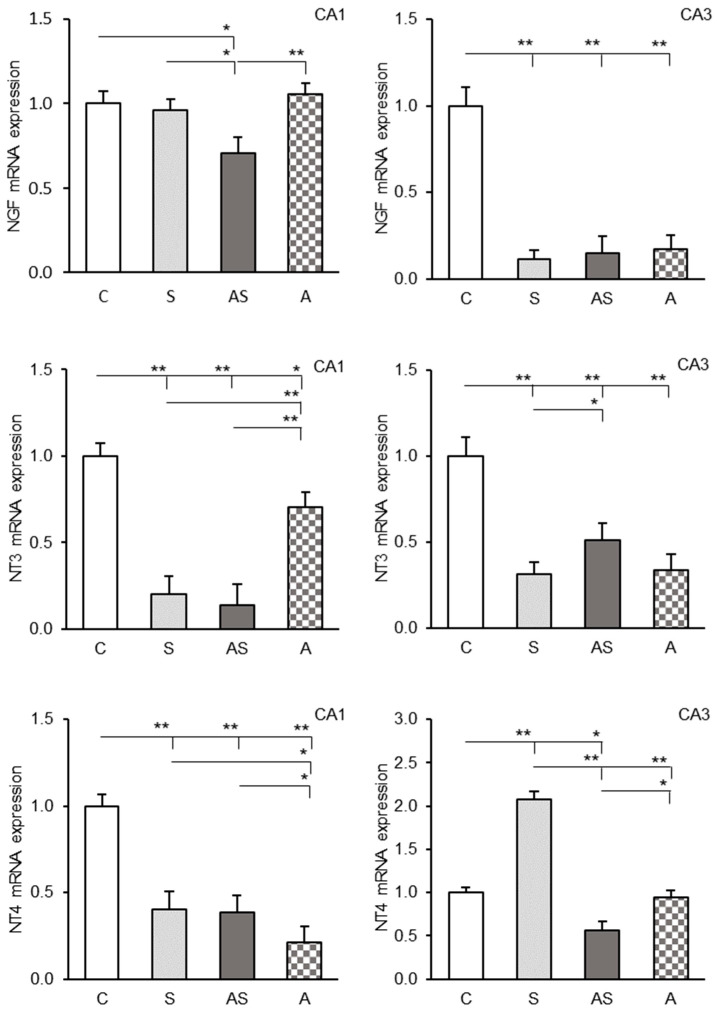
Relative nerve growth factor (NGF, (**top**)), neurotrophin-3 (NT3, (**middle**)) and neurotrophin-4 (NT4, (**bottom**)) mRNA expression (mean ± SEM) in hippocampal CA1 (**left panel**) and CA3 (**right panel**) fields of sheep treated with control solution (C), control solution and stressful stimuli (S), allopregnanolone (4 × 15 μg/60 μL/30 min/day) and stressful stimuli (AS) and allopregnanolone alone (A). Significance of differences: * *p* < 0.05, ** *p* < 0.01.

In the hippocampal CA1 region, NGF mRNA levels were significantly reduced only in the stress + ALLO group (AS) compared to other groups (*p* < 0.05–*p* < 0.01), (Figure 3, top, left panel). Transcript levels of *NT3* and *NT4* were decreased in all experimental groups (S, A, AS) relative to controls (*p* < 0.05–*p* < 0.01), (Figure 3, middle and bottom, left panel). In addition, the ALLO-only group (A) showed elevated *NT3* (*p* < 0.01) but reduced *NT4* (*p* < 0.05) expression compared to stress-exposed groups (S, AS).

In the CA3 region, all experimental treatments (S, AS, and A groups) resulted in a significant (*p* < 0.01) reduction in NGF mRNA abundance compared to the control group (Figure 3, top, right panel). *NT3* transcripts were similarly decreased (*p* < 0.01), though the AS group maintained higher levels (*p* < 0.05) than the S group (Figure 3, middle, right panel). For NT4, there was a significant (*p* < 0.01) increase in mRNA expression in the stressed sheep (S) compared to the control and the AS and A groups (Figure 3, bottom, right panel). Conversely, ALLO infusion combined with stress exposure (AS) caused a significant reduction (*p* < 0.05) in NT4 mRNA levels in the CA3 compared to the control and A groups, while infusion of ALLO alone (A) had no significant effect on NT4 mRNA levels.

## 3. Discussion

The present study demonstrated that acute 4 h stress predominantly suppressed neurotrophic activity in the sheep hippocampus. This was evidenced by reductions in BDNF protein levels and decreased mRNA expression of BDNF, TrkB, as well as most other neurotrophins in the hippocampal CA1 and CA3 fields. Moreover, the findings indicated that neurotrophin and TrkB receptor expression could be modulated by ALLO, a neurosteroid involved in the central stress response mechanism [6,20,21]. Intracerebroventricular ALLO administration lowered both BDNF protein concentrations and BDNF/TrkB transcript levels in hippocampal tissue.

Sheep, as herd animals, possess a strong social instinct, and experience significant psychological distress when isolated from their herds. Our earlier study demonstrated that a 4 h isolation period combined with restricted movement effectively activated the hypothalamic–pituitary–adrenal (HPA) axis at all functional levels [24]. This neuroendocrine system plays a pivotal role in response to unpredictable environmental stressors, facilitating adaptation to changing conditions [25]. Our findings revealed substantial activation of corticotropin-releasing hormone (CRH) neuronal activity in the paraventricular nucleus (PVN) in response to the described stimuli, as evidenced by elevated *CRH* and *arginine vasopressin* (*AVP*) gene transcript levels and the release of CRH into the extracellular spaces of the infundibular nucleus/median eminence. Consequently, there was also a marked surge in adrenocorticotropic hormone (ACTH) secretion from the anterior pituitary, resulting in elevated plasma cortisol levels [24]. Neuroendocrine changes in sheep exposed to stressors were accompanied by a specific behavioral pattern. A particularly strong stress factor for the sheep was the experience of isolation from the flock, which induced severe anxiety and a desire to escape from the cage, accompanied by loud vocalization. In the majority of cases, this behavior persisted throughout the entire 4 h experiment. It should be noted that all experimental sheep were in the luteal phase, characterized by relatively stable blood progesterone levels [26]. Progesterone is known to enhance BDNF synthesis and secretion in various CNS regions. A growing body of evidence indicates that many neuroprotective effects of this steroid, particularly observed in experimental models focusing on the hippocampus and cortex, are mediated through neurotrophins [27].

Considering the neurohormonal context of our ovine model, a significant decrease in *BDNF* gene expression and its protein levels was observed in the hippocampal CA1 and CA3 regions following acute stress exposure. This decrease was accompanied by a reduction in mRNA transcript for TrkB, a high affinity BDNF receptor, and altered expression patterns of other neurotrophins. Current research concerning the effects of stress on BDNF expression has been mainly conducted in rodent models. Studies have shown that social isolation reduces BDNF expression in various brain regions in adult mice and rats [28,29], while acute restraint stress decreases BDNF mRNA and protein in the hippocampus [30,31]. Interestingly, a more detailed analysis showed that acute restraint initially increases hippocampal BDNF mRNA within the first 60 min before subsequent decline [32]. Moreover, elevated levels of BDNF peptide persisted 180 min after stress exposure, but returned to baseline levels after 300 min. This pattern suggests that similar temporal dynamics may have occurred in our sheep model, as experimental groups maintained relatively high BDNF protein levels despite significant reductions compared to controls. Notably, stress exposure induced distinct neurotrophin responses, including elevated NT4 mRNA in CA3 and stable *NGF* transcript levels in CA1, potentially reflecting their protective roles in stress adaptation. As previously proposed [32], rapid neurotrophin upregulation may represent a compensatory mechanism to preserve hippocampal homeostasis and/or modulate stress-related neurotransmitter systems. This is particularly relevant for BDNF, which is known to increase glutamatergic transmission and spontaneous calcium oscillation [33,34].

The relationship between CNS levels of neurotrophic factors and HPA axis activity is particularly significant in the hippocampus, where both CA1 and CA3 fields are rich in glucocorticoid receptors (GR) and mineralocorticoid receptors (MR) [35]. The hippocampus has been implicated in modulating the glucocorticoid response during psychogenic stress involving potential threat perception [36]. Notably, initial stress responses involve concurrent GR activation and BDNF mRNA production in the hippocampus [32,37]. Revest et al. [38] showed that the activation of hippocampal GR induced tissue plasminogen activator expression, which mediates pro-BDNF conversion to mature BDNF, thereby activating BDNF-TrkB signaling. This mechanism is likely part of the aforementioned complex signaling pathway [32] that also enhances stress-related contextual memory formation, facilitating adaptation to acute stressors [38]. The relationship between BDNF and GR appears bidirectional, as BDNF can induce the phosphorylation of GR [39], suggesting reciprocal regulation. Moreover, strong expressions of GR and MR in the hippocampus can also be associated with its key role in HPA axis regulation through descending projections. While most hippocampal projections are stimulatory, evidence suggests that the hippocampus can indirectly inhibit PVN CRH neuron activity via GABAergic neurons in the bed nucleus of the stria terminalis (BNST) [36,40]. Although this pathway may suppress acute stress responses at the hypothalamic level, its involvement in glucocorticoid negative feedback remains uncertain.

Unlike the early stress response, prolonged stress exposure for several hours (from 2 to 8 or longer) in rodents suppresses BDNF expression in both CA1 and CA3 hippocampal fields [41,42]. Reduction in BDNF and TrkB receptor expression after 4 h of stress exposure, observed in the present study, was consistent with findings reported for rodents [41,42], confirming attenuated neuronal excitability and diminished BDNF-TrkB signaling. TrkB receptors are known to be localized in the nerve terminals, axons, and dendritic spines of glutamatergic pyramidal and granule cells of the hippocampus [43]. Upon activation, they can initiate three major signaling cascades, including the Ras-mitogen-activated protein kinase (MAPK) pathway, the phosphatidylinositol 3-kinase (PI3K)-Akt pathway, and the PLCγ-Ca^2+^ pathway [44]. In the hippocampus, BDNF-TrkB signaling primarily mediates synaptic plasticity, driving both structural and functional synaptic modifications. The dominant form of synaptic plasticity is long-term potentiation (LTP), identified as a key cellular mechanism underlying learning and memory [15]. Since BDNF-TrkB signaling plays a key role in LTP modulation [45], prolonged stress exposure may disrupt this signaling, with potentially severe implications for cognitive processes. Specifically, prolonged glucocorticoid exposure induces deleterious structural and cellular alterations in the hippocampus, including dendritic atrophy in CA3 and dentate gyrus (DG) neurons, as well as loss of dendritic spines in CA1 neurons [46,47]. In contrast, Radecki et al. [48] demonstrated that chronic intra-hippocampal infusion of BDNF prevented LTP deficits and preserved learning and memory. Reduced BDNF-TrkB signaling during prolonged stress may also disrupt adult hippocampal neurogenesis. This lifelong process predominantly occurs in the subgranular zone (SGZ) of the hippocampal DG and the subventricular zone (SVZ) of the lateral ventricles, and involves proliferation, differentiation, and migration of neural precursors to form new neurons [49,50]. The existence of neurogenic niches in the SGZ and the SVZ has also been confirmed in sheep, with evidence indicating that adrenal steroids exert an inhibitory effect on the proliferation of newly formed cells [51]. Research demonstrates that the survival and synaptic integration of newborn neurons are tightly regulated by neurotrophic factors. However, this process can be disrupted by several mechanisms, including decreased neurotrophic signaling due to acute or prolonged stress exposure, expression of the BDNF Val66Met variant (impairing BDNF secretion), and TrkB receptor deletion in hippocampal progenitors [52,53,54]. Interestingly, the present study found that the expression of certain neurotrophins in the sheep hippocampus resisted prolonged stress, and was maintained at a constant level (NGF in the CA1) or even increased (NT4 in the CA3). Other studies show that unpredictable and temporal alterations in neurotrophic factors at the CNS level may predispose them to the development of stress-associated pathology like psychiatric and neurodegenerative disorders. For example, major depressive disorder is associated with impaired hippocampal plasticity, neuronal atrophy, synaptic depression, accompanied by reduced plasma BDNF levels [55,56].

Research has indicated that hippocampal neurons and glial cells possess the complete set of enzymes necessary for neurosteroid synthesis from cholesterol [1,2]. Like in other brain regions, increased hippocampal neurosteroid production, especially ALLO, is associated with the HPA axis response to acute stress. The precise mechanism driving ALLO synthesis during stress remains unclear, but its primary functions are to restore homeostasis by dampening HPA axis hyperactivity via the inhibitory GABAergic system [23]. The GABA_A_ receptor complex is the primary target of ALLO, mediating its inhibitory effects through GABA potentiation or direct activation of GABA currents [6,7,8]. Studies in rodents and sheep have shown that ALLO reduces corticotropic neuropeptide gene expression and release, contributing to its anti-stress and anxiolytic effects [24,57]. The neuronal trans-synaptic pathway inhibiting HPA axis activity primarily involves BNST-derived GABAergic neurons [36,40], whereas hippocampal GABAergic systems form local inhibitory circuits regulating various aspects of cellular function [58]. It cannot be ruled out that ALLO, in interaction with this local population of GABAergic neurons, may contribute to the temporary inhibition of local BDNF expression and release. Our findings suggest that periodic ALLO elevations in cerebrospinal fluid or brain structures may suppress BDNF-TrkB signaling and partially inhibit other neurotrophin pathways. Notably, ALLO alone was more effective than stress exposure in reducing BDNF protein in the CA3 field and *NT4* transcript in the CA1 field. Conversely, the neurosteroid had no significant effect on NGF mRNA expression in the CA1 field or NT4 mRNA in the CA3 field. Similar results were obtained earlier by Naert et al. [59], who observed a transient 25% decrease in hippocampal BDNF levels 30 min after intraperitoneal ALLO injection in rats, followed by a significant increase after 180 min. Importantly, the sheep’s brain structures were excised exactly 30 min after the last infusion of allopregnanolone. It is therefore likely that we have captured this initial suppression phase of BDNF secretion in our large animal model. Interestingly, the combined stress + ALLO treatment elicited distinct neurotrophin expression patterns in the CA1 and CA3 compared to either factor alone, suggesting different sensitivity of these two hippocampal fields to the experimental factors. As mentioned earlier, acute stress causes a transient increase in hippocampal BDNF expression and levels in the initial phase [32], which is associated with neuronal excitation [15]. The observed suppression of neurotrophic activity by ALLO may serve an important protective function, potentially preventing over-stimulation of hippocampal excitatory neurons during the critical phase of acute stress. The potential involvement of hippocampal GABAergic circuits in mediating this effect requires further investigation. Additionally, the observed effects may involve other independent stress response pathways operating at genomic and non-genomic levels [60]. Notably, stress counteracted the inhibitory effect of the administered allopregnanolone dose on BDNF release in the CA3 region, highlighting the strength of the emotional impact.

## 4. Materials and Methods

### 4.1. Animal Management

The experiment utilized 24 Polish Longwool sheep, a breed characterized by seasonal reproduction, aged 2 years and weighing 55 ± 2 kg. The animals were housed at the Sheep Breeding Center of the Kielanowski Institute of Animal Physiology and Nutrition, Polish Academy of Sciences (52° N, 21° E) under natural lighting conditions. Throughout the experiment, the sheep were kept indoors in individual pens and fed twice a day with pelleted concentrate diet according to the recommendations of the National Research Institute of Animal Production (Krakow-Balice, Poland) and the National Institute for Agricultural Research (France) [61]. Hay, water, and mineral licks were available ad libitum.

### 4.2. Brain Surgery

One month prior to the experiment, the sheep underwent the stereotaxic implantation of a stainless-steel guide cannula (outer diameter: 1.2 mm) into the third ventricle (IIIv) of the brain. The surgery was performed under general anesthesia induced by intravenous administration of xylazine (40 mg/kg body weight) and xylapan combined with ketamine (10 to 20 mg/kg body weight) (Bioketan, Vetoquinol Biowet, Pulawy, Poland), following the procedure described by Traczyk and Przekop [62]. The cannula was positioned at the frontal coordinate of 31.0 mm, according to the stereotaxic coordinate system designed for the sheep hypothalamus [63]. It was fixed to the skull with stainless steel screws and dental cement, and the external opening of the canal was closed with a stainless-steel cap. After the surgery, the sheep received daily antibiotic injections for five days (1 g streptomycin and 1,200,000 IU benzylpenicillin; Polfa, Warszawa, Poland) and analgesics for four days (metamizole sodium 50 mg/animal; Biovetalgin, Biowet Drwalew, Drwalew, Poland), or meloxicam (1.5 mg/animal; Metacam, Boehringer Ingelheim, Ingelheim am Rhein, Germany). Cannula placement in the IIIv was intraoperatively verified by cerebrospinal fluid outflow and confirmed postmortem. All sheep used in the present study had correctly positioned cannulae.

### 4.3. Experimental Design and Tissue Collection

The experiment was conducted during the reproductive season (mid-October to mid-December) using estrus-synchronized sheep. Estrus was induced using the Chronogest-CR method (Intervet, Boxmeer, The Netherlands), with PMSG administration (500 IU, Folligon, Intervet, Boxmeer, The Netherlands) triggering behavioral estrus within 24–36 h [24]. During the late luteal phase (days 12–14) of the subsequent cycle, animals were randomly allocated to four treatment groups (n = 6/group): (i) intracerebroventricular (icv.) infusion of a vehicle for 3 days (C group); (ii) icv. infusion of a vehicle for 3 days with exposure to stressful stimuli for 4 h on day three (S group); (iii) icv. infusion of ALLO for 3 days with exposure to stressful stimuli for 4 h on day three (AS group); and (iv) icv. infusion of ALLO alone for 3 days (A group). All icv. infusions were administered in a series of four 30 min infusions, at 30 min intervals (from 10:00 to 14:00 h), using a BAS Bee microinjection pump and calibrated 1.0 mL gas-tight syringes (Bioanalytical Systems Inc., West Lafayette, IN, USA). The ALLO dosage (4 × 15 μg/60 μL/30 min/day) was determined based on preliminary experiment (unpublished data, Grant No. 2015/19/B/NZ9/03706). The compound was reconstituted in a solution of DMSO (Blirt, DNA Gdańsk, Gdańsk, Poland) and 20% 2-hydroxypropyl-β-cyclodextrin (1:1) (Sigma-Aldrich, Saint Luis, MO, USA) 24 h before infusion and then diluted using Ringer–Locke (RL) solution before administration [24]. The control animals received an identical vehicle solution without ALLO. Each portion of the infusion mixture contained 2% DMSO. This intermittent infusion protocol, previously validated in our ovine model [24,64], maintains bioactive concentrations while preventing continuous receptor saturation. During the trial, the sheep were kept in pairs in the experimental room in comfortable cages, ensuring normal resting behavior, to which they had been previously adapted for at least 2 days.

On the third day of the experiment, stress was induced through 4 h of social isolation (removal of companion sheep) combined with partial movement restriction (preventing escape from the cage) during infusion (4 h). This combination of stressors was previously validated in our sheep model to reliably induce a pronounced stress response of the HPA axis [24]. Directly after the experiment, the animals received an intravenous anesthetic cocktail (xylazine 0.2 mg/kg + ketamine 3 mg/kg body weight) (Bioketan, Vetoquinol Biowet, Pulawy, Poland) prior to euthanasia in a dedicated slaughter facility. The brains were promptly removed from the skull immediately after slaughter. Following separation of the median eminence, each brain was sagittally sectioned into cerebral hemispheres. The hippocampus was dissected from the medial part of the temporal lobe of the right hemisphere, as described previously [65]. Subsequently, 2 mm sections of the hippocampal CA1 and CA3 fields were cut. All sectioning was performed on sterile glass plates kept on ice, and the collected structures were flash-frozen in liquid nitrogen and then stored at −80 °C until analysis.

### 4.4. Tissue BDNF Concentration Assay

Frozen sections of the hippocampal CA1 and CA3 fields were mixed with radioimmunoprecipitation assay buffer (0.5 M Tris-HCl, pH 7.4, 1.5 M NaCl, 2.5% deoxycholic acid, 10% NP-40, 10 mM EDTA) (Merck, Darmstadt, Germany) in a 1:10 tissue-to-buffer ratio, along with the protease inhibitor aprotinin (10 IU/mL, Sigma-Aldrich, Saint Louis, MO, USA). The samples were homogenized using a laboratory homogenizer and ceramic beads, as described previously [66]. BDNF concentration in the homogenates was determined using the Biosensis Mature BDNF Rapid ELISA kit (BEK-2211, Biosensis Pty Ltd., Thebarton, Australia) according to the manufacturer’s protocol. Although originally designed for humans, mice, and rat samples, this ELISA kit has been validated for measuring mature BDNF in biological samples from other mammals, including sheep [66]. The assay demonstrated high reproducibility with intra- and interassay coefficients of variation (CVs) of 1% and 5%, respectively, and a detection sensitivity of less than 2 pg/mL. The total protein content in the tissue homogenates was determined spectrophotometrically using the Bradford method and the Bio-Rad Protein Assay Kit II (Bio-Rad, Hercules, CA, USA) following the manufacturer’s instructions. BDNF concentrations in each homogenate sample were normalized to total protein and reported as picograms per milligram of protein.

### 4.5. Neurotrophin mRNA Abundance Assay

Total RNA from hippocampal tissues was isolated using the NucleoSpin RNA II kit (Macherey-Nagel, Düren, Germany), according to the manufacturer’s protocol. RNA concentration and purity were quantified using a NanoDrop ND-1000 spectrophotometer (Thermo Fisher Scientific, Waltham, MA, USA). RNA integrity was verified electrophoretically on a 1.5% agarose gel stained with ethidium bromide. Complementary DNA (cDNA) was synthesized using the TranScriba Kit (A&A Biotechnology, Gdynia, Poland) with 1 µg of total RNA in a 20 µL reaction volume, following the manufacturer’s instructions. A quantitative polymerase chain reaction (qPCR) was conducted using 5× HOT FIREPol^®^ EvaGreen qPCR Mix Plus (Solis BioDyne, Tartu, Estonia) as described previously [66]. Specific primers for the expression analysis of *BDNF*, *NGF*, *NT3*, *NT4,* and *TrkB* genes, along with the reference genes glyceraldehyde-3-phosphate dehydrogenase (*GAPDH*) and peptidylprolyl isomerase C (*PPIC*), were designed using Primer3 v. 4.1.00 software (The Whitehead Institute, Boston, MA, USA) (Table 1). Amplification specificity was further validated by electrophoresis of the PCR amplicons on a 2% agarose gel and visualized under a UV light. Expression data were analyzed using Rotor Gene 6000 v. 1.7 software (Qiagen, Hilden, Germany) employing a comparative quantification option and the Relative Expression Software Tool (REST 2009 v. 1), based on the PCR efficiency correction algorithm developed by Pfaffl et al. [67,68]. Gene expression levels were normalized to the geometric mean of reference gene expressions. Endogenous controls were included in all runs to account for variations in cDNA input and PCR efficiency.

### 4.6. Statistical Analysis

Initially, data normality was assessed using the Shapiro–Wilk test, and subsequently, the data were grouped into parametric and non-parametric groups. One-way analysis of variance, followed by the post hoc least significance difference test (STATISTICA v. 13.3, Stat Soft, Tulsa, OK, USA) was used to evaluate differences in tissue BDNF concentrations between treatment groups. For mRNA expression levels of neurotrophins and the TrkB receptor in the hippocampal fields, statistical analysis was conducted using the Kruskal–Wallis test, followed by pairwise group comparisons with the Mann–Whitney U test. Statistical significance was set at *p* < 0.05, and results are expressed as mean ± standard error of the mean (SEM).

## 5. Conclusions and Limitations

The study demonstrated that a 4 h acute stress exposure transiently inhibited the synthesis of BDNF, TrkB, and several other neurotrophins in the sheep hippocampus. Furthermore, ALLO, whose increased levels are highly correlated with the initial stress response, may serve as a mediator of this stress effect, temporarily preventing over-stimulation of hippocampal BDNF release and signaling. However, there are a few limitations to the experiment that require clarification. The experiment used a single dose of ALLO that was selected on the basis of previous preliminary study. Although the effectiveness of individual stressors may vary, removing sheep from the flock (a psychological stressor) can undoubtedly mask the effects of partial movement restriction (a physical stressor). This was evidenced by the behavior of animals and the accompanying HPA axis parameters described earlier [24]. These stressors were combined out of necessity to prevent the animal from escaping from the experimental cage during isolation. The established paradigm complicates conducting an in-depth statistical analysis to demonstrate the additive, synergistic, or independent effects of the factors. However, the changes in neurotrophic activity observed in this study under the influence of ALLO, may provide a basis for understanding some of the processes previously described in our sheep model [24,65,69].

## Figures and Tables

**Table 1:** Specific primers used for gene expression analysis.

Gene	Primers (5′–3′)	Genbank Acc. No.	Amplicon Size
*BDNF*	F: CGTTGGCTGACACTTTTGAA R: CGCAGCATCCAGGTAATTTT	XM_012143442.1	188
*NGF*	F: CAGTCCAAGGGGCTGGAT R: AGTGTGGCCAGGACAGAAAG	XM_004002369.5	101
*NT3*	F: TGCCACGATCTTACAGGTGA R: TGCCTGGATCAGCTTGATTA	XM_004006944.5	151
*NT4*	F: CCTGAGATGTCACGAAGGAC R: TGAACACCTGTCAGCACCTC	XM_027978595.3	112
*TRKB*	F: TGTCTGAGCTGATCCTGGTG R: TATCTGCAGGTTTGCCAGTG	XM_012117231.2	155
*GAPDH*	F: GGGTCATCATCTCTGCACCT R: GGTCATAAGTCCCTCCACGA	NM_001190390.1	131
*PPIC*	F: TGGAAAAGTCGTGCCCAAGA R: TGCTTATACCACCAGTGCCA	XM_004008676.1	158

*BDNF:* brain-derived neurotrophic factor, *NGF*: nerve growth factor, *NT3*: neurotrophin-3, *NT4*: neurotrophin-4, *TrkB*: tyrosine kinase receptor B, *GAPDH*: glyceraldehyde-3-phosphate dehydrogenase, *PPIC*: peptidylprolyl isomerase C, F: forward primer, R: reverse primer. Real-time PCR amplification efficiency of target and reference genes was 96–100%.

## Data Availability

The datasets analyzed during the current study are available from the corresponding author upon reasonable request.

## Abbreviation

The following abbreviations are used in this manuscript:

ACTH	Adrenocorticoid hormone
ALLO	Allopregnanolone
AVP	Arginine vasopressin
BNST	Bed nucleus of the stria terminalis
BDNF	Brain-derived neurotrophic hormone
CA	Cornu ammonis (1 and 3)
CNS	Central nervous system
CRH	Corticotropin-releasing hormone
DG	Dentate gyrus
GABA	Gamma-aminobutyric acid
GAPDH	Glyceraldehyde-3-phosphate dehydrogenase
GR	Glucocorticoid receptors
HPA	Hypothalamic–pituitary–adrenal
LTP	Long-term potentiation
MR	Mineralocorticoid receptors
NGF	Nerve growth factor
NT3	Neurotrophin 3
NT4	Neurotrophin 4
P75NTR	P75 neurotrophin receptor
PPIC	Peptidylprolyl isomerase C
PVN	Paraventricular nucleus
RL	Ringer–Locke solution
SGZ	Subgranular zone
SVZ	Subventricular zone
Trk	Tropomyosin receptor kinases
